# Supplementary material for: Pyruvate Dehydrogenase Kinase 1 inhibition mediated oxidative phosphorylation enhancement in cartilage promotes osteoarthritis progression
Source: BMC Musculoskelet Disord. 2023 Jul 20;24:597. doi: 10.1186/s12891-023-06585-6 (PMC10357736; doi:10.1186/s12891-023-06585-6)
Supplement: Supplementary file 1 — Additional file 1. [file 12891_2023_6585_MOESM1_ESM.zip › KEGG.Gsea.1653622432464/KEGG_P53_SIGNALING_PATHWAY.html]

Details for gene set KEGG\_P53\_SIGNALING\_PATHWAY[GSEA]

|  || Dataset | 55457\_collapsed\_to\_symbols.cls.cls #OA\_versus\_normal.cls.cls #OA\_versus\_normal\_repos |
| Phenotype | cls.cls#OA\_versus\_normal\_repos |
| Upregulated in class | normal |
| GeneSet | KEGG\_P53\_SIGNALING\_PATHWAY |
| Enrichment Score (ES) | -0.31205663 |
| Normalized Enrichment Score (NES) | -1.2620538 |
| Nominal p-value | 0.11582382 |
| FDR q-value | 0.30045623 |
| FWER p-Value | 1.0 |
Table: GSEA Results Summary

  

Fig 1: Enrichment plot: KEGG\_P53\_SIGNALING\_PATHWAY      
 Profile of the Running ES Score & Positions of GeneSet Members on the Rank Ordered List

  

| SYMBOL | TITLE | RANK IN GENE LIST | RANK METRIC SCORE | RUNNING ES | CORE ENRICHMENT || 1 | ADGRB1 | adhesion G protein-coupled receptor B1 [Source:HGNC Symbol;Acc:HGNC:943] | 655 | 0.605 | -0.0200 | No |
| 2 | CDKN2A | cyclin dependent kinase inhibitor 2A [Source:HGNC Symbol;Acc:HGNC:1787] | 799 | 0.570 | -0.0018 | No |
| 3 | STEAP3 | STEAP3 metalloreductase [Source:HGNC Symbol;Acc:HGNC:24592] | 1011 | 0.515 | 0.0082 | No |
| 4 | ZMAT3 | zinc finger matrin-type 3 [Source:HGNC Symbol;Acc:HGNC:29983] | 1169 | 0.483 | 0.0207 | No |
| 5 | CDK4 | cyclin dependent kinase 4 [Source:HGNC Symbol;Acc:HGNC:1773] | 1375 | 0.447 | 0.0277 | No |
| 6 | CD82 | CD82 molecule [Source:HGNC Symbol;Acc:HGNC:6210] | 2056 | 0.356 | -0.0070 | No |
| 7 | RCHY1 | ring finger and CHY zinc finger domain containing 1 [Source:HGNC Symbol;Acc:HGNC:17479] | 2234 | 0.335 | -0.0036 | No |
| 8 | IGF1 | insulin like growth factor 1 [Source:HGNC Symbol;Acc:HGNC:5464] | 2286 | 0.328 | 0.0093 | No |
| 9 | CCNB1 | cyclin B1 [Source:HGNC Symbol;Acc:HGNC:1579] | 2359 | 0.319 | 0.0201 | No |
| 10 | SIAH1 | siah E3 ubiquitin protein ligase 1 [Source:HGNC Symbol;Acc:HGNC:10857] | 2389 | 0.315 | 0.0340 | No |
| 11 | CCNE2 | cyclin E2 [Source:HGNC Symbol;Acc:HGNC:1590] | 2469 | 0.306 | 0.0436 | No |
| 12 | CCND2 | cyclin D2 [Source:HGNC Symbol;Acc:HGNC:1583] | 2508 | 0.302 | 0.0561 | No |
| 13 | CHEK2 | checkpoint kinase 2 [Source:HGNC Symbol;Acc:HGNC:16627] | 2533 | 0.299 | 0.0696 | No |
| 14 | CHEK1 | checkpoint kinase 1 [Source:HGNC Symbol;Acc:HGNC:1925] | 3173 | 0.233 | 0.0319 | No |
| 15 | CASP3 | caspase 3 [Source:HGNC Symbol;Acc:HGNC:1504] | 3202 | 0.230 | 0.0415 | No |
| 16 | DDB2 | damage specific DNA binding protein 2 [Source:HGNC Symbol;Acc:HGNC:2718] | 3257 | 0.225 | 0.0488 | No |
| 17 | RRM2 | ribonucleotide reductase regulatory subunit M2 [Source:HGNC Symbol;Acc:HGNC:10452] | 3505 | 0.202 | 0.0400 | No |
| 18 | CCNG1 | cyclin G1 [Source:HGNC Symbol;Acc:HGNC:1592] | 3711 | 0.184 | 0.0335 | No |
| 19 | CDK1 | cyclin dependent kinase 1 [Source:HGNC Symbol;Acc:HGNC:1722] | 3744 | 0.182 | 0.0403 | No |
| 20 | FAS | Fas cell surface death receptor [Source:HGNC Symbol;Acc:HGNC:11920] | 3828 | 0.173 | 0.0428 | No |
| 21 | GADD45A | growth arrest and DNA damage inducible alpha [Source:HGNC Symbol;Acc:HGNC:4095] | 4081 | 0.148 | 0.0307 | No |
| 22 | PPM1D | "protein phosphatase, Mg2+/Mn2+ dependent 1D [Source:HGNC Symbol;Acc:HGNC:9277]" | 4159 | 0.142 | 0.0320 | No |
| 23 | CCND3 | cyclin D3 [Source:HGNC Symbol;Acc:HGNC:1585] | 4426 | 0.119 | 0.0174 | No |
| 24 | TP53I3 | tumor protein p53 inducible protein 3 [Source:HGNC Symbol;Acc:HGNC:19373] | 4516 | 0.112 | 0.0162 | No |
| 25 | TP53AIP1 | tumor protein p53 regulated apoptosis inducing protein 1 [Source:HGNC Symbol;Acc:HGNC:29984] | 4540 | 0.110 | 0.0201 | No |
| 26 | IGFBP3 | insulin like growth factor binding protein 3 [Source:HGNC Symbol;Acc:HGNC:5472] | 5167 | 0.059 | -0.0257 | No |
| 27 | SESN1 | sestrin 1 [Source:HGNC Symbol;Acc:HGNC:21595] | 5204 | 0.056 | -0.0256 | No |
| 28 | TP73 | tumor protein p73 [Source:HGNC Symbol;Acc:HGNC:12003] | 5222 | 0.055 | -0.0241 | No |
| 29 | CCNG2 | cyclin G2 [Source:HGNC Symbol;Acc:HGNC:1593] | 5358 | 0.043 | -0.0324 | No |
| 30 | TP53 | tumor protein p53 [Source:HGNC Symbol;Acc:HGNC:11998] | 5511 | 0.032 | -0.0427 | No |
| 31 | CCNE1 | cyclin E1 [Source:HGNC Symbol;Acc:HGNC:1589] | 5663 | 0.018 | -0.0535 | No |
| 32 | BID | BH3 interacting domain death agonist [Source:HGNC Symbol;Acc:HGNC:1050] | 5715 | 0.014 | -0.0567 | No |
| 33 | ATM | ATM serine/threonine kinase [Source:HGNC Symbol;Acc:HGNC:795] | 6179 | -0.019 | -0.0918 | No |
| 34 | PERP | p53 apoptosis effector related to PMP22 [Source:HGNC Symbol;Acc:HGNC:17637] | 6324 | -0.030 | -0.1015 | No |
| 35 | TSC2 | TSC complex subunit 2 [Source:HGNC Symbol;Acc:HGNC:12363] | 6946 | -0.075 | -0.1460 | No |
| 36 | MDM2 | MDM2 proto-oncogene [Source:HGNC Symbol;Acc:HGNC:6973] | 7273 | -0.101 | -0.1662 | No |
| 37 | CCND1 | cyclin D1 [Source:HGNC Symbol;Acc:HGNC:1582] | 7512 | -0.119 | -0.1786 | No |
| 38 | EI24 | EI24 autophagy associated transmembrane protein [Source:HGNC Symbol;Acc:HGNC:13276] | 7582 | -0.126 | -0.1775 | No |
| 39 | SFN | stratifin [Source:HGNC Symbol;Acc:HGNC:10773] | 8118 | -0.164 | -0.2108 | No |
| 40 | CASP9 | caspase 9 [Source:HGNC Symbol;Acc:HGNC:1511] | 8142 | -0.166 | -0.2040 | No |
| 41 | CDK6 | cyclin dependent kinase 6 [Source:HGNC Symbol;Acc:HGNC:1777] | 8393 | -0.185 | -0.2140 | No |
| 42 | CYCS | "cytochrome c, somatic [Source:HGNC Symbol;Acc:HGNC:19986]" | 8410 | -0.186 | -0.2057 | No |
| 43 | GADD45G | growth arrest and DNA damage inducible gamma [Source:HGNC Symbol;Acc:HGNC:4097] | 8671 | -0.206 | -0.2154 | No |
| 44 | BAX | "BCL2 associated X, apoptosis regulator [Source:HGNC Symbol;Acc:HGNC:959]" | 9323 | -0.257 | -0.2529 | No |
| 45 | PMAIP1 | phorbol-12-myristate-13-acetate-induced protein 1 [Source:HGNC Symbol;Acc:HGNC:9108] | 10084 | -0.329 | -0.2951 | Yes |
| 46 | SERPINE1 | serpin family E member 1 [Source:HGNC Symbol;Acc:HGNC:8583] | 10284 | -0.349 | -0.2927 | Yes |
| 47 | CASP8 | caspase 8 [Source:HGNC Symbol;Acc:HGNC:1509] | 10442 | -0.366 | -0.2861 | Yes |
| 48 | CCNB2 | cyclin B2 [Source:HGNC Symbol;Acc:HGNC:1580] | 10639 | -0.387 | -0.2816 | Yes |
| 49 | TNFRSF10B | TNF receptor superfamily member 10b [Source:HGNC Symbol;Acc:HGNC:11905] | 11020 | -0.433 | -0.2889 | Yes |
| 50 | MDM4 | MDM4 regulator of p53 [Source:HGNC Symbol;Acc:HGNC:6974] | 11047 | -0.437 | -0.2685 | Yes |
| 51 | CDK2 | cyclin dependent kinase 2 [Source:HGNC Symbol;Acc:HGNC:1771] | 11069 | -0.441 | -0.2475 | Yes |
| 52 | ATR | ATR serine/threonine kinase [Source:HGNC Symbol;Acc:HGNC:882] | 11231 | -0.461 | -0.2364 | Yes |
| 53 | THBS1 | thrombospondin 1 [Source:HGNC Symbol;Acc:HGNC:11785] | 11498 | -0.501 | -0.2314 | Yes |
| 54 | RPRM | "reprimo, TP53 dependent G2 arrest mediator homolog [Source:HGNC Symbol;Acc:HGNC:24201]" | 11727 | -0.538 | -0.2215 | Yes |
| 55 | PTEN | phosphatase and tensin homolog [Source:HGNC Symbol;Acc:HGNC:9588] | 11836 | -0.556 | -0.2014 | Yes |
| 56 | PIDD1 | p53-induced death domain protein 1 [Source:HGNC Symbol;Acc:HGNC:16491] | 12172 | -0.636 | -0.1949 | Yes |
| 57 | BBC3 | BCL2 binding component 3 [Source:HGNC Symbol;Acc:HGNC:17868] | 12298 | -0.676 | -0.1699 | Yes |
| 58 | CDKN1A | cyclin dependent kinase inhibitor 1A [Source:HGNC Symbol;Acc:HGNC:1784] | 12727 | -0.890 | -0.1575 | Yes |
| 59 | APAF1 | apoptotic peptidase activating factor 1 [Source:HGNC Symbol;Acc:HGNC:576] | 12812 | -0.993 | -0.1131 | Yes |
| 60 | GADD45B | growth arrest and DNA damage inducible beta [Source:HGNC Symbol;Acc:HGNC:4096] | 12816 | -1.001 | -0.0619 | Yes |
| 61 | GTSE1 | G2 and S-phase expressed 1 [Source:HGNC Symbol;Acc:HGNC:13698] | 12894 | -1.332 | 0.0005 | Yes |
Table: GSEA details [plain text format]

  

Fig 2: KEGG\_P53\_SIGNALING\_PATHWAY      
 Blue-Pink O' Gram in the Space of the Analyzed GeneSet

  

Fig 3: KEGG\_P53\_SIGNALING\_PATHWAY: Random ES distribution      
 Gene set null distribution of ES for **KEGG\_P53\_SIGNALING\_PATHWAY**
